# Exposure to Photoperiod-Melatonin-Induced, Sexually-Activated Rams after Weaning Advances the Resumption of Sexual Activity in Post-Partum Mediterranean Ewes Lambing in January

**DOI:** 10.3390/vetsci4010004

**Published:** 2017-01-21

**Authors:** José A. Abecia, Philippe Chemineau, Andrea Gómez, Carlos Palacios, Matthieu Keller, José A. Delgadillo

**Affiliations:** 1 Instituto Universitario de Investigación en Ciencias Ambientales (IUCA), Departamento de Producción Animal y Ciencia de los Alimentos, Universidad de Zaragoza, Miguel Servet, 177, Zaragoza 50013, Spain; andreagg1992@hotmail.com; 2 Physiologie de la Reproduction et des Comportements, Institut National de la Recherche Agronomique, Centre National De La Recherche Scientifique (INRA, CNRS), Université de Tours, Agreenium, Nouzilly 37380, France; philippe.chemineau@tours.inra.fr (P.C.); matthieu.keller@tours.inra.fr (M.K.); 3 Departamento de Construcción y Agronomía, Facultad de Ciencias Agrarias y Ambientales, Filiberto Villalobos, Salamanca 37007, Spain; carlospalacios@colvet.es; 4 Centro de Investigación en Reproducción Caprina, Universidad Autónoma Agraria Antonio Narro, Periférico Raúl López Sánchez y Carretera a Sante Fe, C.P. 27054, Torreón, Coahuila, Mexico; joaldesa@yahoo.com

**Keywords:** ram, photoperiod treatment, melatonin, anestrus

## Abstract

This study was aimed to determine whether the presence of sexually stimulated rams by photoperiodic and melatonin treatments can advance the resumption of post-partum sexual activity in Mediterranean ewes lambing in January and weaned at the end of the breeding season at 41°N, in March. Rams were exposed to two months of long days (16 h light/day) and given three melatonin implants at the end of the long days (sexually-activated rams; SAR). Control rams (CR) were exposed to the natural photoperiod. Thirty-six ewes weaned on 25 February were assigned to one of two groups. From 1 March to 30 June, one group was housed with four SAR males (SAR-treated; *n* = 18), and the other group (CR-treated; *n* = 18) was housed with four unstimulated rams. Ovulation was assessed once per week based on plasma progesterone concentrations. Estrus was monitored daily by marks left on ewes by rams’ harnesses. SAR-treated ewes had a shorter (*p* < 0.01) weaning–first estrus interval than CR-treated ewes (61 ± 17 days vs. 102 ± 47 days; mean date of first estrus after weaning on April 26 and June 6, respectively). The proportion of the ewes ovulating in April or May was higher (*p* < 0.05) in the SAR-treated group than in the CR-treated group. SAR-treated ewes resumed estrous activity sooner than CR-treated ewes such that, in April, May, and June, the proportion of females that exhibited estrus was higher (*p* < 0.01) in the SAR-treated group (72%, 89%, and 100%, respectively) than in the CR-treated group (17%, 44%, and 61%, respectively). In conclusion, the introduction at weaning of sexually activated rams advanced the resumption of estrous activity in ewes in spring. The practical implications of this work could be important in ewes adapted for intensive production and accelerated lambing systems.

## 1. Introduction

The photoperiod is the main environmental factor that influences the seasonality of reproduction in sheep [1]; however, other natural environmental factors such as nutrition, temperature, and the presence of conspecifics can have a modest influence on the timing of the onset and the offset of seasonal reproduction [2,3,4]. Seasonality in reproduction ensures that births occur at the optimal time of the year; usually, at the end of winter or the beginning of spring, which allows the newborn to survive and grow under favorable conditions of temperature and food availability [5]. The anestrous period covers the late winter/early spring to early- or mid-summer period, whilst the transition period expands from late spring to the onset of the ovulatory period. This seasonal breeding pattern results in a clear period of lambing, which in turn causes a seasonal pattern of product prices with prices being lowest when the supply of meat is the highest (late spring to early fall) and vice versa. If farmers were able to produce products “out of-season”, they could take advantage of higher prices for these during the winter by inducing estrous cycles during the seasonal anestrus. 

In Mediterranean sheep breeds, the onset is earlier and the offset of the breeding season is later than they are in breeds that live at more northern latitudes [6]; consequently, Mediterranean breeds have a shorter seasonal anestrus (i.e., three months from April through June in the Rasa Aragonesa breed [7]) than do Northern European breeds (i.e., 7 months from March through September in the Scottish Blackface breed [8]). In addition, those females respond better to management practices such as the ram effect or the strategic use of feed supplements, which shorten the periods of reproductive inactivity [9].

In ewes, the postpartum anestrus in spring coincides with the onset of the seasonal anestrus because lactation and the increase in day length coincide. Induction of estrus and pregnancy during seasonal anestrus permits lambing during the breeding season and consequently, the next mating can occur shortly after weaning [10]. After late-winter or spring lambing, however, the postpartum anestrus is longer, and ewes are unable to resume cyclic activity after weaning [11]. Thus, length of postpartum anestrus varies greatly depending on latitude and timing of the lambing season [12,13]. The presence of a mature male during the postpartum period shortens the interval between parturition and the first ovulation. In cattle, cows exposed to bulls early in the postpartum period had reduced intervals between calving and the resumption of ovarian activity and first behavioral estrus. They resumed cyclicity with estrous cycles of normal length, and had more estrous periods in the 90 days prior to the breeding season than did cows not exposed to bulls [14,15]. In sheep, and only when lambing occurred during the breeding season, the continuous presence of rams with the ewes from the start of the lambing period advanced the resumption of postpartum ovarian activity by approximately 30 days and caused a peak in estrus about 40 days after lambing [16]. In Awassi sheep, however, at the time of spring rebreeding, postpartum ewes did not respond to the continuous presence of rams [17], and the length of the interval between lambing and ram introduction dictated the effectiveness of stimulation by the presence of rams in autumn-lambing Barbarine ewes [18]. 

Recently, we demonstrated that ewes continuously exposed to sexually active rams that had been stimulated by an artificial photoperiod and melatonin implants maintained their ovarian activity in spring, which resulted in an increase in estrous expression [19]. Moreover, the presence of photoperiodic and melatonin treatment-induced, sexually-activated rams in spring advanced puberty in autumn-born ewe lambs, providing an effective and sustainable means of increasing the productive life of ewes while avoiding the use of hormonal treatments [20]. Given the capacity of light-treated rams to prolong reproductive activity of ewes in spring, and the effect of exposure to males on the induction of ovarian activity during the breeding season in the Rasa Aragonesa breed, we hypothesized that the presence of rams that had been sexually stimulated by specific photoperiodic treatments could advance the resumption of sexual activity in ewes that lamb in winter (January) and wean at the end of the breeding season, in March. This breed, which is representative of the “Mediterranean” sheep breeds, exhibits a three-month anestrous season between April and July, although some (10%–45%) ewes ovulate in spring [7]. The practical implications of this work could be important in the reactivation of estrous activity and ovulation rate in ewes adapted for intensive production and accelerated lambing systems. 

## 2. Material and Methods

### 2.1. Experimental Procedure

**Figure 1:**
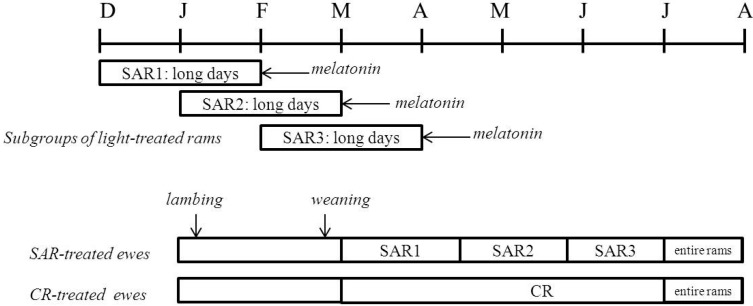
Experimental design used in the experiment. Sexually-activated rams (SAR) were exposed to two months of long days (16 h of light/day) and three subcutaneous melatonin implants at the end of the long-day period, when were returned to natural photoperiod. They were exposed to long days between 1 December and 31 January (SAR), between 1 January and 28 February (SAR2), or between 1 February and 31 March (SAR3). The groups received subcutaneous melatonin implants on 1 February (SAR1), 1 March (SAR2), or 1 April (SAR3). Control rams (CR) (*n* = 4) were exposed to natural photoperiodic conditions. Thirty-six ewes lambing in the first week of January and weaned on 25 February were divided into two groups, which were allocated to different shaded, open pens. From 1 March, one group (SAR-treated group) was housed with two photoperiodic treatment-stimulated SAR1 rams; SAR2 rams replaced SAR1 rams on 14 April, and SAR3 rams replaced SAR2 rams on 22 May, and remained with the ewes until 30 June. The other group of ewes (CR-treated group) was housed with the unstimulated rams. Four Rasa Aragonesa rams were introduced to each of the ewe groups on 1 July and remained until 31 July.

Light + melatonin treatment of rams and the protocol of exposing ewes to rams are similar to those described by our group for the activation of puberty of ewe lambs [20] (Figure 1).

### 2.2. Sexual Activation of Rams

The experiment included 16 vasectomized, sexually experienced adult Rasa Aragonesa rams of 5–8 years of age; live weight (LW, mean ± standard error of mean (S.E.M.): 105 ± 3 kg; body condition score (BCS) [21]: 3.25 ± 0.0). Control rams (*n* = 4) were kept in a shaded, open pen and exposed to natural photoperiodic conditions (15 h and 12 min, and 9 h and 10 min of light, at the summer and winter solstices, respectively). Photoperiodic-treated rams (*n* = 12) were kept permanently in a shaded, open pen under natural photoperiodic conditions before the photoperiodic treatments, which consisted of two months of long days (16 h of light/day) in a closed pen (5 m × 7 m; 8.75 m^2^/ram) and three subcutaneous melatonin implants (Melovine, CEVA Salud Animal, Barcelona, Spain) at the end of the long-day period, when rams were returned to natural photoperiod conditions. Lighting was controlled by an electronic timer and light intensity was at least 300 lx at the level of the eyes of the animals. To ensure that ewes were exposed continuously to sexually-activated rams (SAR) throughout the experiment, treated rams were assigned to one of three groups. The ram groups were exposed to long days between December 1 and January 31 (SAR1 group, *n* = 4), the second group was exposed to long days between January 1 and February 28 (SAR2 group, *n* = 4), and the third group was exposed to long days between February 1 and March 31 (SAR3 group, *n* = 4) (Figure 1). The groups received subcutaneous melatonin implants on February 1 (SAR1), March 1 (SAR2), or April 1 (SAR3). Artificial light was provided in the morning (6:00 to 09:00) and at night (16:00 to 22:00). Light intensity was at least 300 lx at the level of the eyes of the animals [22]. We have demonstrated that this combination of artificial photoperiod and melatonin treatment stimulates testosterone secretion in Rasa Aragonesa rams in spring, differing significantly between the treated and the control rams [20]. 

### 2.3. Females

The experiment included 36 Rasa Aragonesa ewes (LW: 57 ± 4 kg; BCS: 2.62 ± 0.12) that lambed in the first week of January (January 5 ± 4 days). At weaning on 25 February (mean lactation length 50 ± 4 days), they were divided into two groups, which were allocated to different shaded, open pens. From 1 March, the sexually-activated ram group (SAR-treated; *n* = 18) was housed with two photoperiodic treatment-stimulated SAR1 rams. SAR2 rams replaced SAR1 rams on 14 April, which were replaced by SAR3 rams on 22 May and remained with the ewes until 30 June. The control ram group (CR-treated; *n* = 18) was housed with the unstimulated CR rams throughout the experiment (1 March–30 June). Both SAR and CR rams were managed in the same manner such that ewes were housed with two males, which were rotated every two weeks with the other two rams in the same group. To prevent a “novel male” effect on ewes when the treated rams in the same group were rotated, or when SAR2/SAR3 rams replaced SAR1/SAR2 rams, they were housed in pens adjacent to those of the females, and rams were separated from ewes by an openwork metal barrier only which allowed visual, olfactory, and nose-to-nose contact between the sexes. The new rams were previously placed waiting to be used in pens adjacent to those of the females about one month before introducing them. The two groups of ewes were housed in different barns that were separated by at least 300 m. Four entire Rasa Aragonesa rams were introduced to each of the ewe groups on July 1 and remained until 31 July (Figure 1). The date of first estrus of those ewes that were not detected in estrus by vasectomized rams was estimated as date at lambing minus 145 days, which is the presumed date of the first estrus. Rams and ewes were fed to meet their live weight maintenance requirements [23] and had unlimited access to water and mineral salts. 

The Rasa Aragonesa sheep is a local breed from northern Spain, whose reproductive characteristics have been described [7]. Rams of the breed exhibit a clear seasonal variation in sperm characteristics [24], and melatonin treatments can stimulate sexual activity [25,26]. 

### 2.4. Measurements

Blood samples were collected weekly by jugular venipuncture from 1 March to 30 June, to determine ovarian activity through plasma progesterone concentrations (P_4_). Samples were centrifuged at 3500× *g* for 30 min and the plasma stored at −20 °C until analysis. Concentration of plasma P_4_ higher than 0.50 ng/mL is indicative of a previous ovulation. To facilitate the daily monitoring of estrus, rams wore marking harnesses, which left colored marks on the rumps of the ewes. Therefore, estrous behavior was monitored daily by identifying the marks left by the harness. Day of the first ovulation after weaning was deemed as the day when the first sample indicative of ovulation was observed. 

### 2.5. Hormonal Assays

P_4_ concentrations were assayed using an ELISA kit for ovine plasma (Ridgeway Science, St. Briavels, Gloucestershire, UK), following the manufacturer’s instructions [27]. The sensitivity was 0.27 ng/mL. Intra-assay and inter-assay coefficients of variation for sample pools of 0.5 and 1 ng/mL were 8.5% and 9.9%, and 12% and 15%, respectively. 

### 2.6. Statistical Analysis

The proportions of the ewes that ovulated or entered estrus were calculated for each month, and the statistical significance of the differences between the SAR and CR-treated groups was compared using a chi-square Test or a Fisher´s exact probability test, as appropriate. A ewe was in estrus or ovulating in a given month if it had at least one estrus/ovulation. Differences between the two ewe groups in the lengths of the intervals lambing-first estrus and weaning-first estrus (mean ± S.D.) were tested by ANOVA. 

### 2.7. Ethical Note

The experiment was conducted at the experimental farm of the University of Zaragoza, Spain (41°40’ N 0°53’ W). The Ethics Committee for Animal Experiments at the University of Zaragoza approved all of the procedures performed in the study (Ethical approval code: 229395). The care and use of animals were in accordance with the Spanish Policy for Animal Protection RD1201/05, which meets the European Union Directive 2010/63 on the protection of animals used for experimental and other scientific purposes.

## 3. Results

The interval weaning-first ovulation did not differ significantly (*p* > 0.05) between the SAR- and CR-treated groups (Table 1). In contrast, the proportion of ewes that ovulated was significantly (*p* < 0.05) higher in the SAR-treated group than it was in the CR-treated group in April and May (Table 2). The number of silent ovulations before the first estrus after weaning was significantly (*p* < 0.01) higher in the CR-treated group than it was in the SAR-treated group (Table 1).

The interval weaning-first estrus was significantly (*p* < 0.01) shorter in the SAR-treated group than it was in the CR-treated group (Table 1). In addition, the proportions of ewes exhibiting estrus from April to June were significantly (*p* < 0.0001) higher in the SAR-treated group than they were in the CR group (Table 2).

## 4. Discussion

The presence of photoperiodic-treated, sexually active rams in spring advanced the resumption of estrous activity in ewes after weaning and, most importantly, occurred during the seasonal anestrous period of the Rasa Aragonesa breed (April to June [7]). Ewes exposed to treated rams resumed sexual activity at the end of April, unlike ewes in the CR-treated group which exhibited their first estrus at the beginning of June, the onset of the breeding season of this breed described at this latitude. In addition, the proportions of ewes that ovulated in April and May were higher in the SAR-treated group than they were in the CR-treated group. The length of the interval between weaning and the resumption of estrous activity differed from that of ewes exposed to unstimulated rams [28,29]. In that study, the mean date of the first detected estrus in ewes that weaned in April or July was 20 July and 28 August, respectively, and the duration of the intervals weaning-first detected estrus was 88 and 64 days, respectively. Collectively, our results support the hypothesis that the presence of sexually active rams advances the resumption of sexual activity in ewes weaned at the end of the breeding season because the resumption of sexual activity occurred during the seasonal anestrous.

The practical benefit of the positive response to the presence of activated rams is more apparent given that our attempts to advance the resumption of ovarian activity in spring by changing the energy or protein content of the diet were unsuccessful. Thus, a higher experimental increase in the level of nutrition in protein and energy after weaning did not advance ovarian or estrous activities in Rasa Aragonesa ewes that lambed in late spring [28,30]. The advancement of the resumption of the sexual activity caused by the presence of sexually active rams might have been due to the intense sexual behavior displayed by males. In two recent studies conducted by our research group on the caprine species in Mexico [31] and Rasa Aragonesa sheep in Spain [19,20], the presence of photo-stimulated, sexually active males allowed ovulations to occur year-round in goats, and prolonged estrous activity in spring in ewes and advanced puberty in autumn-born ewe lambs in spring, We suspect that the stimulation of the pulsatile activity of Gonadotropin-releasing hormone /luteinizing hormone (GnRH/LH) in females caused by the presence of sexually active males is the underlying process that caused males to prevent the seasonal inhibition of GnRH/LH pulsatility, which normally occurs at the end of winter or in early spring when females are among sexually inactive males or isolated from sexually active males. Therefore, based on our present study, we hypothesize that the presence of photostimulated rams, which display intense sexual behavior and probably odor, was important in advancing the timing of sexual activity in ewes. If that is true, the practical benefits are most applicable to the anestrus-lambing ewe, which is the most difficult category of ewe in which to induce pregnancy because it has both lactational and seasonal anestrus [32]. 

In our study, the onset of ovarian activity in the absence of estrous signals, detected through plasma P_4_ concentrations, was similar in the SAR- and CR-treated groups, and was shortly after the weaning date. Most of the ewes had several silent ovulations before their first detected estrus, which was most frequent in the CR-treated group. In our previous study that assessed whether the presence of sexually active rams that had been stimulated by artificial photoperiod reduced seasonal anestrus, the frequency of silent ovulations was high among Rasa Aragonesa ewes that were exposed to control rams [19], probably because of the sexual activity of these rams. In several sheep breeds, rams tend to exhibit some sexual activity, even during the natural period of seasonal rest [31]. As in our previous work, in the current study the experiment did not include a group that had no rams in the flock; therefore, we suspect that the residual sexual activity of the rams, and their own pheromone signal [33] contributed to the relatively high proportion of ewes in the control group that ovulated. Moreover, to ensure that SAR-ewes had continuous exposure to sexually-activated rams, three groups of photoperiodic-treated rams were used in the experiment, since rams become insensitive or refractory to melatonin after about 16 weeks of exposure [34]. Probably, the control rams were not sufficiently active to stimulate the GnRH/LH processes that are necessary to induce ovulatory activity and the full expression (and detection) of estrous activity in the ewes, although the fact that the same four rams were used in this group could have affected the response. Note that, in those experiments, the ram was a treatment factor and a diagnostic tool, since estrus was detected based on ram marks. Probably, the diagnostic power of the two ram groups differed because they did not have the same level of sexual activity, which might have led to an underestimation of estrous activity in the control group [19]. Unfortunately, treated rams cannot be used to detect estrous behavior in control ewes because their presence would stimulate these ewes. None of the ewes in the present experiment exhibited estrus immediately after weaning, which occurs in French and British sheep breeds that lamb within the breeding season [10,35], or in Rasa Aragonesa ewes [28].

## 5. Conclusions

In conclusion, the introduction at weaning of sexually activated rams advanced the resumption of estrous activity in ewes in spring, which might be an effective procedure in lamb production systems in which ewes are bred twice yearly or three times in two years, when the interval between lambing and rebreeding should be shorter than is typical in the breed. Ewes that produce lambs late in the breeding season or in early spring give birth at a time when they normally enter seasonal anestrus, and the procedure described in the present experiment can be used to avoid an overlap between postpartum and seasonal anestrus.

## Figures and Tables

**Table 1:** Mean (± S.D.) lambing date, duration of lactation (days), date of first ovulation, date of first estrus, interval weaning-ovulation, interval weaning-estrus (days), and number of silent ovulations before the first estrus in Rasa Aragonesa ewes housed with sexually-activated rams (SAR; two months of 16 h of light/day plus melatonin implants) or control rams (CR), from 1 March to 30 June.

	SAR-Treated	CR-Treated
	*n* = 18	*n* = 18
Lambing date	January 5 ± 4	January 6 ± 6
Weaning	February 25	February 25
Duration lactation (days)	51 ± 4	52 ± 6
First ovulation	March 21 ± 13	March 23 ± 26
Interval weaning-ovulation (days)	26 ± 13	24 ± 26
First estrus	April 26 ± 17 **^a^**	June 6 ± 47 **^b^**
Interval weaning-estrus (days)	61 ± 17 **^a^**	102 ± 47 **^b^**
Number of ovulations before 1st estrus	2.2 ± 1.5 **^a^**	4.5 ± 3.1 **^b^**

a, b: *p* < 0.01.

**Table 2:** Proportions (%) of the Rasa Aragonesa ewes weaned on 25 February that exhibited ovulation and estrus that were housed with sexually-activated rams (SAR; two months of 16 h of light/day plus melatonin implants) or control rams (CR), from 1 March to 30 June.

	March		April		May		June	
	SAR	CR	SAR	CR	SAR	CR	SAR	CR
Ewes ovulating	14/18	13/18	18/18 **^a^**	14/18 **^b^**	18/18 **^a^**	14/18 **^b^**	18/18	18/18
	78%	72%	100%	78%	100%	78%	100%	100%
Ewes in estrus	0/18	2/18	13/18 **^c^**	3/18 **^d^**	16/18 **^c^**	8/18 **^d^**	18/18 **^c^**	11/18 **^d^**
	0%	11%	72%	17%	89%	44%	100%	61%

Within months a, b: *p* < 0.05; c, d: *p* < 0.01.
